# Supplementary material for: Multi‐dataset identification of innovative feature genes and molecular mechanisms in keratoconus
Source: J Cell Mol Med. 2024 Sep 19;28(18):e70079. doi: 10.1111/jcmm.70079 (PMC11412914; doi:10.1111/jcmm.70079)
Supplement: Supplementary file 2 — Material S2. [file JCMM-28-e70079-s002.zip › 7.GSEA analysis/5.RNF217-AS1/KEGG_BASAL_CELL_CARCINOMA.html]

Details for gene set KEGG\_BASAL\_CELL\_CARCINOMA[GSEA]

|  || Dataset | RNF217-AS1.group.cls#h\_versus\_l |
| Phenotype | group.cls#h\_versus\_l |
| Upregulated in class | l |
| GeneSet | KEGG\_BASAL\_CELL\_CARCINOMA |
| Enrichment Score (ES) | -0.56372684 |
| Normalized Enrichment Score (NES) | -1.6851407 |
| Nominal p-value | 0.009727626 |
| FDR q-value | 0.35238138 |
| FWER p-Value | 0.429 |
Table: GSEA Results Summary

  

Fig 1: Enrichment plot: KEGG\_BASAL\_CELL\_CARCINOMA      
 Profile of the Running ES Score & Positions of GeneSet Members on the Rank Ordered List

  

| SYMBOL | TITLE | RANK IN GENE LIST | RANK METRIC SCORE | RUNNING ES | CORE ENRICHMENT || 1 | WNT9A | na | 2856 | 0.297 | -0.0500 | No |
| 2 | WNT9B | na | 4560 | 0.231 | -0.0740 | No |
| 3 | WNT8A | na | 5716 | 0.192 | -0.0864 | No |
| 4 | PTCH2 | na | 5918 | 0.186 | -0.0719 | No |
| 5 | WNT3 | na | 9757 | 0.090 | -0.1728 | No |
| 6 | GSK3B | na | 10962 | 0.071 | -0.1998 | No |
| 7 | WNT2 | na | 11310 | 0.066 | -0.2027 | No |
| 8 | FZD9 | na | 12064 | 0.055 | -0.2185 | No |
| 9 | TCF7L2 | na | 12940 | 0.043 | -0.2390 | No |
| 10 | HHIP | na | 13129 | 0.040 | -0.2401 | No |
| 11 | DVL1 | na | 14350 | 0.026 | -0.2724 | No |
| 12 | GLI1 | na | 14354 | 0.026 | -0.2696 | No |
| 13 | WNT3A | na | 14964 | 0.019 | -0.2850 | No |
| 14 | AXIN1 | na | 15329 | 0.016 | -0.2938 | No |
| 15 | DVL3 | na | 15800 | 0.011 | -0.3062 | No |
| 16 | WNT4 | na | 16250 | 0.006 | -0.3185 | No |
| 17 | TCF7L1 | na | 16424 | 0.004 | -0.3230 | No |
| 18 | WNT1 | na | 17572 | -0.007 | -0.3553 | No |
| 19 | FZD5 | na | 18663 | -0.017 | -0.3849 | No |
| 20 | WNT10A | na | 19364 | -0.024 | -0.4025 | No |
| 21 | FZD6 | na | 19485 | -0.025 | -0.4032 | No |
| 22 | CTNNB1 | na | 19818 | -0.028 | -0.4097 | No |
| 23 | TCF7 | na | 19939 | -0.029 | -0.4100 | No |
| 24 | WNT7B | na | 19942 | -0.029 | -0.4068 | No |
| 25 | SUFU | na | 20159 | -0.031 | -0.4096 | No |
| 26 | WNT7A | na | 21895 | -0.050 | -0.4543 | No |
| 27 | PTCH1 | na | 22222 | -0.053 | -0.4579 | No |
| 28 | GLI3 | na | 22253 | -0.053 | -0.4530 | No |
| 29 | GLI2 | na | 23043 | -0.062 | -0.4689 | No |
| 30 | WNT10B | na | 23653 | -0.070 | -0.4789 | No |
| 31 | WNT2B | na | 24447 | -0.082 | -0.4928 | No |
| 32 | BMP2 | na | 25185 | -0.093 | -0.5039 | No |
| 33 | WNT5A | na | 25202 | -0.094 | -0.4942 | No |
| 34 | FZD10 | na | 25265 | -0.095 | -0.4857 | No |
| 35 | STK36 | na | 26094 | -0.109 | -0.4977 | No |
| 36 | DVL2 | na | 26784 | -0.122 | -0.5044 | No |
| 37 | FZD1 | na | 27779 | -0.143 | -0.5175 | No |
| 38 | WNT8B | na | 29287 | -0.184 | -0.5409 | No |
| 39 | WNT16 | na | 30080 | -0.209 | -0.5410 | Yes |
| 40 | BMP4 | na | 30086 | -0.209 | -0.5184 | Yes |
| 41 | SMO | na | 30221 | -0.214 | -0.4990 | Yes |
| 42 | FZD4 | na | 30972 | -0.241 | -0.4944 | Yes |
| 43 | FZD3 | na | 31028 | -0.243 | -0.4695 | Yes |
| 44 | APC | na | 31337 | -0.256 | -0.4505 | Yes |
| 45 | FZD8 | na | 31536 | -0.265 | -0.4274 | Yes |
| 46 | WNT6 | na | 31862 | -0.283 | -0.4060 | Yes |
| 47 | TP53 | na | 32227 | -0.304 | -0.3834 | Yes |
| 48 | FZD7 | na | 33284 | -0.383 | -0.3721 | Yes |
| 49 | FZD2 | na | 33686 | -0.431 | -0.3368 | Yes |
| 50 | SHH | na | 33702 | -0.433 | -0.2901 | Yes |
| 51 | LEF1 | na | 34024 | -0.487 | -0.2464 | Yes |
| 52 | APC2 | na | 34163 | -0.517 | -0.1941 | Yes |
| 53 | WNT5B | na | 34219 | -0.533 | -0.1377 | Yes |
| 54 | WNT11 | na | 34469 | -0.631 | -0.0762 | Yes |
| 55 | AXIN2 | na | 34629 | -0.769 | 0.0029 | Yes |
Table: GSEA details [plain text format]

  

Fig 2: KEGG\_BASAL\_CELL\_CARCINOMA      
 Blue-Pink O' Gram in the Space of the Analyzed GeneSet

  

Fig 3: KEGG\_BASAL\_CELL\_CARCINOMA: Random ES distribution      
 Gene set null distribution of ES for **KEGG\_BASAL\_CELL\_CARCINOMA**
